# Noninvasive Prenatal Screening Based on Second-Trimester Ultrasonographic Soft Markers in Low-Risk Pregnant Women

**DOI:** 10.3389/fgene.2021.793894

**Published:** 2021-12-23

**Authors:** Yunyun Liu, Xiaosha Jing, Lingling Xing, Sha Liu, Jianlong Liu, Jing Cheng, Cechuan Deng, Ting Bai, Tianyu Xia, Xiang Wei, Yuan Luo, Quanfang Zhou, Qian Zhu, Hongqian Liu

**Affiliations:** ^1^ Medical Genetics Department/Prenatal Diagnostic Center, West China Second University Hospital, Sichuan University, Chengdu, China; ^2^ Key Laboratory of Birth Defects and Related Diseases of Women and Children, Ministry of Education, Sichuan University, Chengdu, China

**Keywords:** noninvasive prenatal screening, ultrasonographic soft markers, trisomy 21 (Down syndrome), sex chromosome abnormality, positive predictive value, aneuploidy

## Abstract

**Background:** We aimed to assess the clinical application of noninvasive prenatal screening (NIPS) based on second-trimester ultrasonographic soft markers (USMs) in low-risk pregnant women.

**Methods:** Data of pregnant women between April 2015 and December 2019 were retrospectively analyzed. Pregnant women [age at expected date of confinement (EDC) of <35 years; low risks for trisomy 21 (T21) and trisomy 18 (T18) based on maternal serum screening; presenting second-trimester USMs (7 types)] who successfully underwent NIPS and had available follow-up information were included in our study. Cases with positive NIPS results were prenatally diagnosed. All patients were followed up for 6 months to 2 years after NIPS, and their clinical outcomes were obtained. Subgroup analyses were performed according to the different USMs.

**Results:** NIPS suggested that among a total of 10,023 cases, 37 (0.37%) were at high risk of aneuploidy, including 4 T21, 6 trisomy 13 (T13), and 27 sex chromosome abnormalities (SCA). Ten cases with aneuploidy (0.10%) were confirmed by prenatal diagnosis, consisting of two T21 and eight SCA. The eight fetuses with SCA consisted of one monosomy X, two XXY, one XXXY, one XXX, one XYY, and two mosaicisms. T21 was detected in one fetus with absent or hypoplastic nasal bone and one fetus with echogenic intracardiac focus (EICF). SCA was detected in five fetuses with EICF, two fetuses with multiple soft markers, and one fetus with echogenic bowel. The positive rate of chromosomal aneuploidy was significantly higher in fetuses with absent or hypoplastic nasal bone (6.25 vs. 0.10%, *p* = 0.017), echogenic bowel (3.7 vs. 0.10%, *p* = 0.029), and multiple soft markers (0.678 vs. 0.10%, *p* = 0.045) than in the total fetuses. The positive predictive values (PPVs) of NIPS in these three groups were 100%, 50%, and 100%, respectively. EICF accounted for 93.25% (9,346/10,023) of the study population, whereas the PPV of NIPS was only 20%.

**Conclusion:** NIPS is an advanced screening test for low-risk pregnant women. In the 10,023 pregnant women sampled, SCA were more common than autosomal trisomy, and EICF was the most frequent USM but the least predictive aneuploidy. Further aneuploidy evaluation is suggested for low-risk pregnant women whose ultrasound indicates absent or hypoplastic nasal bone, echogenic bowel, or multiple soft markers. NIPS can serve as a second-line complementary screening for these women.

## Introduction

With the rapid development of ultrasound technology for application in the first and second trimesters, an increasing number of small ultrasound markers have been discovered, that is, ultrasonographic soft markers (USMs). USMs have a special ultrasonic feature; some USMs disappear naturally in later pregnancy stages or after delivery, whereas others may persist even after birth. USMs are closely associated with fetal chromosomal abnormalities and adverse pregnancy outcomes. The association between nuchal translucency thickness and Down syndrome was first reported in the 1980s (Benacerraf et al., 1985). Since then, diverse ultrasound anomalies have been reported to be associated with trisomy 21 (T21) (Nyberg and Souter, 2001), such as echogenic intracardiac focus (EICF), absent or hypoplastic nasal bone, and mild pyelectasis. Nevertheless, several studies have shown that USMs increase the incidence of invasive prenatal puncture surgery (Ahman et al., 2014). Lee et al. (2007) also found that the detection and interpretation of USMs were correlated to an increase in maternal anxiety and unnecessary amniocentesis.

Moreover, in the relevant laws, regulations, norms, or corresponding guidelines of China, there are currently no clear provisions on how to handle USMs. Article 17 of the Law of the People’s Republic of China on Maternal and Infant Health Care (revised on August 30, 2018 and effective as of August 30, 2018) stipulates that if doctors find or suspect fetal abnormalities after prenatal examination, prenatal diagnosis should be made for pregnant women. Article 20 of the Measures for the Implementation of Law of the Peoples Republic of China on Maternal and Infant Health Care (promulgated on June 20, 2001) stipulates that if a fetus is abnormal or has suspicious malformations, doctors should make a prenatal diagnosis; however, USMs do not necessarily indicate fetal abnormalities or malformations. To further confirm aneuploidy, most Chinese doctors refer to the consensus issued by the American College of Obstetricians and Gynecologists (ACOG) (American College of Obstetricians and Gynecologists’ Committee on Practice Bulletins-Obstetrics et al., 2020) and the Society for Maternal-Fetal Medicine (SMFM) (Prabhu et al., 2021). ACOG Practice Bulletin 226 (American College of Obstetricians and Gynecologists’ Committee on Practice Bulletins-Obstetrics et al., 2020) states that if aneuploidy testing shows a low-risk result, then no further risk assessment is needed for fetus exhibiting particular USMs such as EICF, choroid plexus cyst, mild pyelectasis, or short femur length. The Society for Maternal–Fetal Medicine (SMFM) (Prabhu et al., 2021) also suggests that fetuses with negative maternal serum screening results for EICF, echogenic bowel, and shortened long bones need no further aneuploidy evaluation. However, only few studies have examined the applicability of these two guidelines for pregnant women in China and the residual risks of NIPS in these women.

Currently, maternal serum screening and NIPS are the major prenatal screening programs for evaluating the risk of aneuploidy. Lo et al. (1997) reported the presence of circulating cell-free fetal DNA (cffDNA) in maternal plasma and serum, which has been rapidly and widely used for prenatal screening owing to its high sensitivity and high positive predictive value (PPV). However, the traditional maternal serum screening method cannot assess the risk of sex chromosome aneuploidy (SCA). Except for Turner syndrome, in which structural anomalies are easily observed, the clinical features of other types of SCA are often undetectable in prenatal ultrasound examinations. Unless SCA is detected by invasive prenatal diagnosis, most fetuses with SCA are not diagnosed until birth or puberty. NIPS *via* massive parallel sequencing enables the detection of fetal SCA. Therefore, NIPS can simultaneously detect the risk of trisomy and SCA.

For the identification of birth defects and for health and economic considerations, prenatal screening methods are applied in China, including maternal serum screening, NIPS, and ultrasound examination. Prenatal screening is usually performed in the following order: first-trimester ultrasound (to detect nuchal translucency, NT), first-trimester serum screening, second-trimester serum screening, and second-trimester ultrasound. According to the relevant regulations in China, NIPS is recommended for pregnant women at intermediate risk based on maternal serum screening. These women are subjected to NIPS at relatively late pregnancy weeks. In contrast, some pregnant women choose NIPS for direct evaluation of fetal aneuploidy after understanding the difference between maternal serum screening and NIPS; these women undergo NIPS at relatively early gestational weeks. Prenatal diagnosis is recommended for pregnant women at high risk based on maternal serum screening and high risk of NIPS, as well as those with advanced age at delivery and an indication of abnormal fetal structure in ultrasound examination.

Previous studies have shown that NIPS has a good ability in detecting aneuploidy, including T21 and SCA, in high-risk populations (Bianchi et al., 2014; Garshasbi et al., 2020). However, the application of NIPS for detecting fetal aneuploidy has not been extensively studied in pregnant women at low risk based on maternal serum screening and follow-up ultrasound indicating USMs. Therefore, we retrospectively reviewed the data of pregnant women who underwent maternal serum screening and NIPS in the Prenatal Diagnosis Center of West China Second University Hospital of Sichuan University. Pregnant women who had low risk for T21 or trisomy 18 (T18) based on maternal serum screening and second-trimester ultrasound and successfully underwent NIPS were included in our study.

The aims of the present study were (1) to assess the clinical application of NIPS in the second trimester in a retrospective cohort of pregnant women with USMs and at low risk for common chromosomal abnormalities according to maternal serum screening; (2) to determine the USMs possessing a strong predictive value in the study population; and (3) to determine the feasibility of recommending the screening method that we designed in clinical genetic counseling from the perspective of health economics.

## Materials and Methods

### Study Population

We retrospectively analyzed the data of pregnant women who underwent maternal serum screening, NIPS, and prenatal diagnosis at the Prenatal Diagnosis Center of West China Second University Hospital of Sichuan University, Chengdu, Sichuan Province, China from April 2015 to December 2019. The inclusion criteria were as follows: (1) age at the expected date of confinement (EDC) of less than 35 years; (2) low risk for T21 and T18 based on maternal serum screening; (3) indication of USMs according to second-trimester ultrasound; and (4) NIPS case. The exclusion criteria were as follows: (1) no NIPS results, including failure during cell-free DNA (cfDNA) extraction and low fetal fraction; (2) no clinical pregnancy outcome, including termination of pregnancy, miscarriage, high-risk NIPS cases who declined further prenatal diagnosis, and cases lost to follow-up.

Pretest counseling was performed by trained clinical geneticists. All patients signed an informed consent form for genetic investigation. The test results were used for research with informed consent from the patients or legal guardians.

## Maternal Serum Screening

This procedure was described in our previous study (Deng et al., 2019; Bai et al., 2021). Risks of fetal T21 and T18 were obtained based on maternal serum screening, and patients were divided into three groups: the low-risk, intermediate-risk, and high-risk groups. A risk value of <1 in 1,000 indicated low risk for T21, and a risk value of <1 in 1,000 suggested low risk for T18. The risk value of the intermediate-risk group was 1/271–1/1,000 for T21 and 1/351–1/1,000 for T18. The risk value of the high-risk group was ≥1/270 for T21 and ≥1/350 for T18.

### First-Trimester and Second-Trimester Ultrasound Examination

First-trimester ultrasound examination for NT and second-trimester ultrasound examination for fetal anomalies were performed in the Department of Diagnostic Ultrasound of West China Second University Hospital, Sichuan University, and other allied hospitals or affiliated hospitals that have medical business cooperation with West China Second Hospital of Sichuan University, following the guidelines of the International Society of Ultrasound in Obstetrics and Gynecology (Salomon et al., 2011). The diagnosis of second-trimester USMs was confirmed by a second experienced ultrasonographer. The following seven types of second-trimester USMs were included in this study: EICF, mild pyelectasis (dilatation of the renal pelvis ≥4 mm), single umbilical artery, mild ventriculomegaly (10 mm and <12 mm), absent or hypoplastic nasal bone (absent or <2.5 mm), echogenic bowel, and short femur length (*Z*-score, −2 to −4).

### Noninvasive Prenatal Screening

Peripheral blood (8–10 ml) was collected from each pregnant woman and then placed into Cell-free BCT tubes (Streck Inc., Omaha, NE, United States). The upper plasma was isolated from the blood samples after being centrifuged twice within 72 h. Fifty microliters of cfDNA was isolated from 1,200 µl of plasma using a DNA extraction kit (Hangzhou Berry Gene Diagnostic Technology Co., Ltd., Hangzhou, China) according to the manufacturer’s instruction. The obtained DNA concentration ranged from 0.05 ng/μl to 0.6 ng/μl. Next, 20 µl of cfDNA libraries was constructed by end filling and adapter ligation, and qPCR analysis was performed to verify whether the concentration and quality of cfDNA libraries were satisfactory. The cfDNA libraries were subjected to massive parallel sequencing using the NextSeq CN500 high-throughput sequencing kit (Illumina) on a NextSeq CN500 platform (Illumina), generating approximately 5 million raw data with 36-bp reads (Liu et al., 2021).

Sequencing reads were uniquely mapped to the hg19 human reference genome. The *Z*-score values of the 24 chromosomes were further calculated using normalized chromosome representation and GC correction (Liu et al., 2021). The fetal autosomal trisomy status was determined based on *Z*-scores (normal range, −3 < *Z* < 3). A high risk of NIPS was defined as a *Z*-score of greater than 3 for chromosome 21, 18, or 13. A *Z*-score value of chromosome 21, 18, or 13 ranging from −3 to 3 indicated a low risk of NIPS. The method for calculating the *Z*-score value of chromosomes X and Y was described in our previous study (Bai et al., 2021).

All participants were given an NIPS test report on the estimated fetal risk of T21, T18, and trisomy 13 (T13) and a supplementary report if a high risk of SCA was suspected. The test was considered a failure if unqualified total cfDNA concentration was obtained twice or if the fetal fraction was twice calculated to be <4%.

### Invasive Prenatal Diagnosis

For pregnant women at high risk of NIPS, clinical counselling was offered by clinical geneticists, and further invasive prenatal diagnosis to determine chromosomal abnormalities was recommended.

Amniocentesis or cordocentesis was performed in the late second trimester or early third trimester, and chromosomal microarray analysis (CMA), copy number variation sequencing (CNV-Seq), or karyotyping was conducted to detect fetal aneuploidy and other fetal chromosome abnormalities, in accordance with previously published studies (Wang et al., 2018; Deng et al., 2019; Hu et al., 2021). Furthermore, all samples were subjected to quantitative fuorescence polymerase chain reaction (QF-PCR) for chromosomes 21, 18, 13, X, and Y, and if the short tandem repeat markers were abnormal, enumeration was performed by fluorescence *in situ* hybridization.

The pregnant women were subjected to clinical follow-up assessments *via* telephone communication and review of medical records from 6 months to 2 years after undergoing NIPS. We also collected data on circumstances before birth, including prenatal diagnosis results, situations of high-risk NIPS cases who declined further prenatal diagnosis, and developmental details diagnosed by pediatricians after birth.

### Statistical Analysis

SPSS Statistics software version 19.0 (SPSS Inc., Chicago, IL, United States) was used for statistical analysis. Comparisons between groups were determined using chi-square test or Fisher’s exact test. Statistical significance was set at *p* < 0.05.

## Results

### Study Participants

**FIGURE 1:**
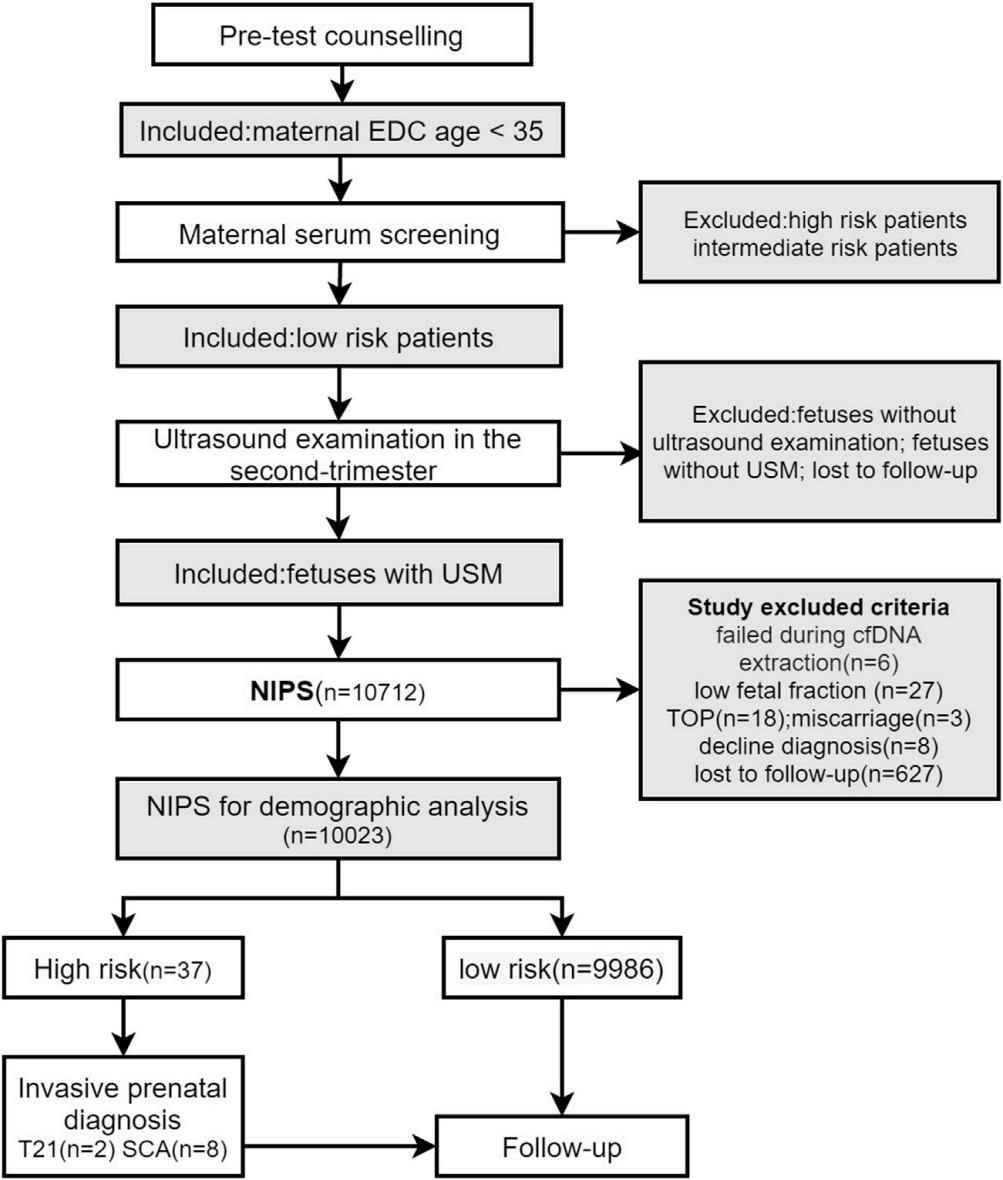
EDC, expected date of confinement; USM, ultrasonographic soft markers; NIPS, noninvasive prenatal screening; TOP, termination of pregnancy.

This was a retrospective study. A total of 143,067 pregnant women underwent NIPS at our hospital between April 2015 and December 2019. A total of 10,023 pregnant women who met the inclusion criteria were included, and retrospective analysis was performed. The study flow diagram is shown in Figure 1.

The maternal age ranged from 16 to 34 years. The median maternal weight was 58.80 kg (range, 37–97 kg), and the median maternal height was 159.00 cm (range, 140–177 cm). The study population predominantly originated from China. A total of 99.60% (9,983/10,023) of the pregnancies were singleton pregnancies, and 1.14% (114/10,023) of the pregnant women conceived with assisted reproductive technology (without Pre-implantation Genetic Testing for Aneuploidy). The demographic characteristics of the pregnant women included in this study are shown in Table 1.

**TABLE 1:** Demographic characteristics of the 10,023 patients.

**Characteristic**	***N* (%)**
Mean maternal age (range), years	26.75 (16–34)
<35 years	10,023 (100%)
Median maternal weight (range), kg	58.80 (37–97)
Median maternal height (range), cm	159.00 (140–177)
Gestational age (range), weeks	24 (14–28)
Race or ethnic group, *n* (%)	
Asian	10,023 (100%)
Singleton pregnancy, *n* (%)	9,983 (99.60%)
Twin pregnancy, *n* (%)	40 (0.40%)
ART pregnancy, *n* (%)	114 (1.14%)

ART, assisted reproductive technology.

EICF was the most common USM present in 9,346 cases (93.25%), followed by multiple soft markers in 295 cases (2.94%) and mild pyelectasis in 166 cases (1.66%).

### Numerical Abnormality of Chromosomes in the Fetus

The overall prevalence of high risk of chromosomal aneuploidy (NIPS-positive results) in the fetuses was 0.37% (37/10,023). Among the 37 cases, 10 fetuses (27.03%) had a high risk of autosomal trisomy, including 4 fetuses at high risk of T21 and 6 fetuses at high risk of T13, and 27 fetuses (72.97%) had a high risk of SCA.

Finally, two cases were confirmed to have T21, and eight fetuses were diagnosed with SCA *via* invasive prenatal diagnosis. As shown in Table 2, among the eight fetuses with SCA, one had monosomy X, two had XXY, one had XXXY, one had XXX, one had XYY, and two had mosaicisms. SCA was detected in two pregnant women with multiple soft markers. Among fetuses with EICF, one T21 and five SCA cases were detected. One T21 case was detected in a fetus with absent or hypoplastic nasal bone, and one SCA was detected in a fetus with echogenic bowel. The other 27 cases showed false-positive results for NIPS, as confirmed by prenatal diagnosis. These cases consisted of 2 fetuses at high risk of NIPS for T21, 6 at high-risk of NIPS for T13, and 19 at high-risk of NIPS for SCA. A false-positive NIPS result for chromosomal aneuploidy was detected in 24 fetuses with EICF, one fetus with mild pyelectasis, one fetus with mild ventriculomegaly, and 1 fetus with echogenic bowel.

**TABLE 2:** Numerical abnormality of chromosomes in the fetus.

Ultrasound category	*n*	High risk of NIPS no	True Positive Validated by Invasive Prenatal diagnosis							Total
			T21	45,X	47,XXY	48,XXXY	47,XXX	47,XYY	Mosaicism	
Multiple soft markers	295	2	—	1	—	1	—	—	—	2
EICF	9,346	30	1		2		1	1	1^a^	6
Mild pyelectasis	166	1	—	—	—	—	—	—	—	0
SUA	117	0	—	—	—	—	—	—	—	0
Mild ventriculomegaly	40	1	—	—	—	—	—	—	—	0
Absent or hypoplastic nasal bone	16	1	1	—	—	—	—	—	—	1
Echogenic bowel	27	2	—	—	—	—	—	—	1^b^	1
Short femur length	16	0	—	—	—	—	—	—	—	0
Total	10,023	37	2 (0.02%)	8 (0.08%)						10 (0.10%)

EICF,echogenic intracardiac focus; SUA, single umbilical artery; T21:trisomy 21.

a NIPS result: high risk of 45,X; diagnosis result: mos 45,X/46,XY, confirmed by FISH: 45,X [15]/46,XY [85].

b NIPS result: high risk of 47,XXX; diagnosis result: mos 47,XXX/46,XX, confirmed by FISH: 47,XXX [77]/46,XX [23].

### Clinical Test Results for NIPS in the Study Population

All NIPS-positive cases were confirmed by prenatal diagnosis and then followed up. No false-negative results were observed in any of the NIPS-negative cases. Thus, the rate of false-negative results for NIPS for T21, T18, T13, and SCA was 0% in this study. The sensitivity, specificity, positive predictive value (PPV), and negative predictive value of NIPS, respectively, were as follows: for T21: 100% (95% CI: 19.79–100%), 99.98% (95% CI: 99.92–99.99%), 50.00% (95% CI: 9.19–90.81%), 100% (95% CI: 99.95–100%); for SCA: 100% (95% CI: 59.77–100%), 99.81% (95% CI: 99.70–99.88%), 29.63% (95% CI: 14.50–50.34%), and 100% (95% CI: 99.95–100%). Among the cases with high risk for T13 as detected by NIPS, there were no true positive cases, resulting in a very low PPV. The clinical test performance of NIPS is outlined in Table 3.

**TABLE 3:** Clinical test performance of NIPS.

Variable	T21	T13	SCA	Total
	(n = 10,023)	(n = 10,023)	(n = 10,023)	(n = 10,023)
Having fetal aneuploidy	—	—	—	—
Test positive for aneuploidy	100.00 (2/2)	0	100.00 (8/8)	100.00 (10/10)
Test negative for aneuploidy	0.00 (0/2)	0	0.00 (0/8)	0.00 (0/10)
Not having fetal aneuploidy	—	—	—	—
Test positive for aneuploidy	0.02 (2/10,021)	0.06 (6/10,023)	0.19 (19/10,015)	0.27 (27/10,013)
Test negative for aneuploidy	99.98 (10,019/10,021)	99.94 (10,017/10,023)	99.81 (9,996/10,015)	99.73 (9,986/10,013)
Sensitivity (95% CI), %	100 (19.79–100)	—	100 (59.77–100)	100 (65.55–100)
Specificity (95% CI), %	99.98 (99.92–99.99)	99.94 (99.86–99.98)	99.81 (99.70–99.88)	99.73 (99.60–99.82)
Positive predictive value (95% CI), %	50.00 (9.19–90.81)	0 (0–48.32)	29.63 (14.50–50.34)	27.03 (14.37–44.39)
Negative predictive value (95% CI), %	100 (99.95–100)	100 (99.95–100)	100 (99.95–100)	100 (99.95–100)
False positive rate (95% CI), %	50.00 (9.19–90.81)	100 (51.68–100)	70.37 (49.66–85.50)	72.97 (55.61–85.63)
False negative rate (95% CI), %	0 (0–0.05)	0 (0–0.05)	0 (0–0.05)	0 (0–0.05)

T21, trisomy 21; SCA, sex chromosome aneuploidy. Data are in percentages with raw numbers shown in parentheses. Statistical analysis shows 95% confidence intervals in parentheses.

### Characteristics of Aneuploidy Among Fetuses With Ultrasonographic Soft Markers

In the study population, the rates of true-positive results for T21 and SCA as confirmed by prenatal diagnosis were 0.02% and 0.08%, respectively. The rate of true-positive results for chromosomal aneuploidy was significantly higher in fetuses with absent or hypoplastic nasal bone (6.25% vs. 0.10%, *p* = 0.017), echogenic bowel (3.7% vs. 0.10%, *p* = 0.029), and multiple soft markers (0.678% vs. 0.10%, *p* = 0.045) than in the total fetuses.

In the multiple soft markers and absent or hypoplastic nasal bone groups, NIPS had a PPV of 100% for aneuploidy, and this value reached 50% in the echogenic bowel group. EICF was the most common and abundant USM, accounting for 93.25% (9,346/10,023) of the study population. In contrast, NIPS had only 20% PPV and 80% false-positive rate in the EICF group. No positive cases were found in the mild and mild ventriculomegaly groups. The results are listed in Table 4.

**TABLE 4:** Characteristics of aneuploidy among the fetuses with USM.

Ultrasound category	*n*	High risk of NIPS, *n*	Diagnosis validated aneuploidy						*p*-Value
			TP	FP	PR(%)	PPV(%)	FPR(%)	FNR(%)	
Multiple soft markers	295	2	2	0	0.678	100	0	0	0.045^a^
EICF	9,346	30	6	24	0.064	20	80	0	—
Mild pyelectasis	166	1	0	1	0	0	100	0	—
SUA	117	0	—	—	—	—	—	—	—
Mild ventriculomegaly	40	1	0	1	0	0	100	0	—
Absent or hypoplastic nasal bone	16	1	1	0	6.25	100	0	0	0.017^b^
Echogenic bowel	27	2	1	1	3.7	50	50	0	0.029^c^
Short femur length	16	0	—	—	—	—	—	—	—
Total	10,023	37	10	27	0.10^d^	27.03^e^	72.97	0	—

EICF, echogenic intracardiac focus; SUA, single umbilical artery; T21, trisomy 21; TP, true positive; FP, false positive; PR, positive rate; PPV, positive predictive value; FPR, false-positive rate; FNR, false-negative rate.

a Positive rate: Multiple soft markers group vs. Total population, *p* = 0.045.

b Positive rate: Absent or hypoplastic nasal bone group vs. Total population, *p* = 0.017.

c Positive rate: Echogenic bowel group vs. Total population, *p* = 0.029.

d The value is 0.59 if the study population excluded the EICF group.

e The value is 50 if the study population excluded the EICF group.

Among the 10 clinically confirmed aneuploidy cases, two pregnant women had a history of spontaneous abortion. Six pregnant women (60%) were selected for prenatal diagnosis with CNV-Seq after positive results for NIPS was obtained. The *Z*-scores of NIPS were >5 in confirmed T21 cases, and the *Z*-scores of X chromosomes were >7 in confirmed Klinefelter syndrome cases. The two pregnant women whose fetus were prenatally diagnosed with mosaicisms chose to give birth. The detailed clinical information is presented in Table 5.

**TABLE 5:** Clinical details of the 10 cases with fetal aneuploidy and positive NIPS.

No	Maternal age	Conception (spontaneous/IVF)	Spontaneous abortion history (Y/N)	Result of maternal serum screening (risk of T21/T18)		USM in second trimester	NIPS positive results	NIPS *Z*-scores	Diagnosis methods	Diagnosis results	Comfirm test	Outcomes
1	29	Spontaneous	Y	1/2,493	1/22,378	EICF	T21	Chr21 12.3	CNV-seq	T21	QF-PCR	TOP
2	25	Spontaneous	N	1/1,496	1/70,695	Absent or hypoplastic nasal bone	T21	Chr21 5.14	CNV-seq	T21	QF-PCR	TOP
3	29	Spontaneous	N	1/6,312	1/35,176	EICF	ChrY+	ChrX −5.91 ChrY 105.41	CNV-seq	47,XYY	FISH	Born
4	28	Spontaneous	N	1/5,221	1/50,000	EICF	ChrX+	ChrX 3.66 ChrY −1.47	CMA	47,XXX	FISH	TOP
5	27	Spontaneous	N	1/13,737	1/100,000	EICF	ChrX+(Y)	ChrX 8.2 ChrY 76.09	CNV-seq	47,XXY	FISH	TOP
6	29	Spontaneous	N	1/50,000	1/50,000	EICF	ChrX-	ChrX −9.52 ChrY 6.02	CNV-seq	Mos 45,X/46,XY	FISH	Born
7	28	Spontaneous	N	1/50,000	1/50,000	EICF	ChrX+(Y)	ChrX 7.14 ChrY 88.36	CMA	47,XXY	FISH	TOP
8	27	IVF	Y	1/22,057	1/100,000	Echogenic bowel	ChrX+	ChrX 10.16 ChrY 0.17	CNV-seq	Mos 47,XXX/46,XX	FISH	Born
9	30	Spontaneous	N	1/11,734	1/97,085	Multiple soft markers (EICF, mild pyelectasis)	ChrX+(Y)	ChrX 12.28 ChrY 57.67	CMA	48,XXXY	FISH	TOP
10	30	Spontaneous	N	1/1,002	1/98,851	Multiple soft markers (EICF,SUA)	ChrX-	ChrX −7.63 ChrY 0.73	CMA	45,X	FISH	TOP

IVF, *in vitro* fertilization; USM, ultrasonographic soft markers; EICF, echogenic intracardiac focus; SUA, single umbilical artery; T21, trisomy 21; Chr,chromosome; CNV-seq, copy number variation sequencing; CMA, chromosomal microarray analysis; FISH, fluorescence *in situ* hybridization; TOP, termination of pregnancy.

### Clinical Follow-Up Outcomes of Noninvasive Prenatal Screening-Negative Cases

The pregnancy outcomes of NIPS-negative pregnant women and fetuses are shown in Table 6. There were 9,752 normal fetuses, accounting for 97.66% of the total NIPS-negative population. There were 234 abnormal cases, accounting for 2.34% of the total NIPS-negative population, among which the fetus with height less than the standard value 2 SD, with weight less than the standard value 2SD, or both, accounted for 1.08% of the total birth population. Preterm births at less than 37 gestational weeks accounted for 0.39%, followed by congenital heart disease. Fetuses delivered by pregnant women with negative NIPS results were evaluated by senior pediatricians, and they reported no T21, T18, T13, SCA, or related abnormalities during neonatal examination and follow-up of this study population.

**TABLE 6:** Pregnancy outcomes in 9,986 women with negative NIPS.

Pregnant outcomes	*n*	Percentage (%)	Remarks
Normal after birth	9,752	97.66	—
Abnormalities	234	2.34	—
Premature delivery (<37 weeks)	39	0.39	—
Neonatal death	6	0.06	Congenital hypopnea syndrome (1 case), congenital heart disease (2 cases), leukemia (1 case), and death from choking on milk (2 cases)
Developmental delay	108	1.08	Height < standard value − 2 SD level or weight < standard value − 2 SD level
Language development delay	5	0.05	—
Congenital heart disease	25	0.25	Including aortic stenosis, tetralogy of Fallot, and atrial septal defect
Autism	2	0.02	—
Harelip	1	0.01	—
Thalassemia	4	0.04	—
Epilepsy	2	0.02	—
Favism	3	0.03	—
Allergy	8	0.08	Milk and egg allergy
Dystonia	5	0.05	1 had low muscle tone and 4 had high muscle tone
Polydactyly	2	0.02	—
Albinism	1	0.01	—
Angioma	8	0.08	—
Congenital valgus deformity of foot	1	0.01	—
Dysplasia of the ear canal and abnormal hearing	11	0.11	Ear canal development malformation (5), and hearing abnormality (6)
Funnel chest	1	0.01	—
Mediastinal tumor of thoracic cavity	1	0.01	—
Langerhans cell histiocytosis	1	0.01	—
Total	9,986	100	—

## Discussion

USMs are of great importance in clinical practice. They are often transient and nonpathological, but may indicate an increased risk of underlying fetal aneuploidy (Hu et al., 2021). In fact, USMs can increase the detection rate of malformations by 4% (Boyd et al., 1998). However, the detection of USMs cause stress in pregnant women, and the depressive symptoms may persist until delivery (Ahman et al., 2010; Nevay et al., 2016). Previous studies have shown that USMs, such as EICF, thickened nuchal fold, and mild pyelectasis were commonly repeated in subsequent pregnancies, providing deeper insights into the genetic predisposition and recurrence of USMs (Ginsberg et al., 2017). In particular, the interpretation of abnormal results and clinical genetic consultation in pregnant women who have had USMs and undergone NIPS may also cause confusion for them and the clinicians. When ACOG and SMFM guidelines are used for genetic counseling, the residual risk in pregnant women in China must be elucidated. Moreover, large-scale studies of the clinical application of NIPS in pregnant women at low risk based on maternal serum screening and second-trimester ultrasound are required. Thus, we designed this study to explore the potential application of NIPS for USM-based detection of fetal aneuploidy in low-risk pregnant women, as well as to formulate a feasible strategy for aneuploidy detection.

The positive rate of aneuploidy was 0.1% (range: 0.064%–6.25%), which suggests a residual risk for aneuploidy of approximately 0.1% in our study population if no further aneuploidy evaluation is conducted. The positive rate of aneuploidy was lower than that reported in previous studies (Norton et al., 2015; Garshasbi et al., 2020; Margiotti et al., 2020). Selection bias might be attributable to this difference. There were several other possible reasons: (1) In our study, we selected patients with age at EDC of <35 years and those with low risk for T21 and T18 based on maternal serum screening, leading to a reduction in the overall background aneuploidy risk in this study population. (2) Several pregnant women whose age at EDC was close to but did not reach 35 years as well as several pregnant women whose maternal serum screening results were very close to the cutoff value may have been more inclined to opt for prenatal diagnosis directly. (3) In the second trimester, T18 and T13 are more likely to show structural abnormalities than T21, and the group of pregnant women who preferred prenatal diagnosis were excluded from our study population.

The mean gestational age in our study was 24 weeks, with a range of 14–28 weeks, and there were differences in the routine timings of NIPS, which is usually performed in the late first trimester or early second trimester (van der Meij et al., 2019). Detailed sonographic anatomical scanning is usually performed at 22–25 weeks of gestation in China. Our study population was pregnant women who underwent NIPS after second-trimester ultrasound examination; thus, the mean gestational age was the second trimester of pregnancy. Previous studies showed that the percentage of fetal fraction significantly increased with increasing gestational age (Hou et al., 2019). Therefore, the detection results of NIPS were credible.

In our study, the sensitivity of NIPS for both T21 and SCA reached 100%, and the specificity was >99.7%. Compared with direct prenatal diagnosis, NIPS delays the diagnosis of aneuploidy, but it can effectively screen fetal aneuploidy. No false-negative cases were found in our study after long-term follow-up (false-negative rate: 0). In addition, false-positive results are a serious issue in NIPS. In our study, 27 false-positive cases were identified. Considering that the cfDNA sample used for NIPS was derived from placenta, and not fetal DNA, the main cause of these false-positive results may be confined placental mosaicism. Other possible causes include vanishing twin syndrome, maternal copy number variants, cancer, previous organ or bone marrow transplantation, medical conditions, or treatment affecting the quality of circulating DNA (Snyder et al., 2015; Bianchi and Chiu, 2018).

In the absence or hypoplastic nasal bone group, a case of T21 was confirmed, with a positive rate of 6.25% (1/16). The highest incidence of T21 was found in fetuses with absent or hypoplastic nasal bone, followed by that in fetuses with EICF. In our study, the PPV of NIPS for T21 was 100% in fetuses in the absent or hypoplastic nasal bone group, which was consistent with the data reported by Du et al. (2018). A previous study has also reported absent or hypoplastic nasal bone as one of the most prominent USMs in the second trimester (Agathokleous et al., 2013).

Isolated EICF was the most common USM in our study. The true-positive rates of T21 and SCA in the EICF group were much lower than those in the other groups. Only one T21 and five SCA cases were confirmed by prenatal diagnosis. This was consistent with previous findings (Agathokleous et al., 2013) that EICF had low positive likelihood ratio values for T21. According to ACOG (Committee on Practice Bulletins-Obstetrics; Committee on Genetics; Society for Maternal-Fetal Medicine. 2020) and SMFM (Prabhu et al., 2021), if isolated EICF is detected, no further risk assessment is needed in pregnant women at low risk based on maternal serum screening. Furthermore, NIPS is a good option for evaluating fetal aneuploidy if the mother demands further aneuploidy assessment. In our total study population, the aneuploidy positive rate and PPV of NIPS were reanalyzed after removing the EICF group, which increased the values to 0.59% and 50%, respectively.

However, large-scale studies focusing on the correlations between the prevalence of SCAs and USM in fetuses in low-risk populations have rarely been published in the literature. In our study, the highest incidence of SCAs was found in fetuses with echogenic bowel (3.7%), followed by those with multiple soft markers (0.68%). In previous studies, echogenic bowel was associated with fetal aneuploidy, congenital infections (mainly congenital CMV infection), structural anomalies (D'Amico et al., 2021; Ronin et al., 2017), the incidence of intrauterine growth restriction and intrauterine fetal demise (Mailath-Pokorny et al., 2012), and Zellweger syndrome (Aydemir et al., 2014). In previous studies, multiple soft markers were associated with a high incidence of chromosomal abnormalities (Wang et al., 2018; Hu et al., 2021). Our study found that the PPV of NIPS and the positive rate of SCAs in multiple soft marker groups were higher than those in the total population.

According to previous studies, *Z*-scores are considered to be correlated with NIPS accuracy (Tian et al., 2018; Zheng et al., 2020). In the current study, the *Z*-scores of NIPS were >5 for T21, and the *Z*-scores of X chromosomes were >7 for Klinefelter syndrome. Thus, in clinical genetic counseling with ultrasound, in addition to different types of USMs, we can also focus on the *Z*-scores of NIPS. Hu et al. (2021) and Wang et al. (2018) showed a correlation between the presence of USMs and the risk of pathogenic copy number variations (pCNVs), particularly in short femur length and multiple soft markers. Furthermore, prenatal diagnosis is recommended for detecting pCNVs in pregnant women with USMs and NIPS positivity. Chromosomal microarray analysis and CNV-Seq are also applicable to these cases.

In follow-up, we found that 17.78% (8/45) of women with positive NIPS results rejected further aneuploidy evaluation. Moreover, in pregnant women with negative results for NIPS, the preterm birth rate was 0.39%, and the proportion of infants with height or weight less than the standard value −2 SD was 1.08% of the overall population. The genetic disorders thalassemia and favism were also identified. Although NIPS can identify fetuses with abnormal chromosome numbers, it is not useful for detecting several monogenic diseases and autosomal recessive genetic disorders. Therefore, pregnant women with a family history of relevant genetic disorders should be fully informed of the scope and limitations of NIPS during genetic counseling.

The large sample size was a considerable strength of our study. Moreover, we performed subgroup analyses of different types of USMs and conducted an in-depth investigation of the applicability of NIPS in fetuses with different types of USMs in a low-risk population. Our findings offer more comprehensive genetic counseling for clinicians when facing a certain type of USMs.

However, our study also had several limitations. First, the distribution of the different types of fetal USMs varied considerably. The number of absent or hypoplastic nasal bone and short femur length cases was relatively small, which might have led to selection bias. Second, the karyotype results remained unknown for cases who had positive results for NIPS but rejected further aneuploidy evaluation, as well as for those who had termination of pregnancy and miscarriage, which might have caused bias in data analysis. Third, the gestational weeks at the time of second-trimester ultrasound were not recorded, and thus we could not incorporate this variable in our analysis.

## Conclusion

Our study suggests that NIPS is an advanced screening test for pregnant women with age at EDC younger than 35 years, a low risk of maternal serum screening, and second-trimester USMs. In our study population, SCA was more common than autosomal trisomy, whereas EICF was the most frequent USM, but the least predictive of aneuploidy. If second-trimester ultrasound indicates absent or hypoplastic nasal bone, echogenic bowel, or multiple USMs, further aneuploidy evaluation is recommended, and NIPS can be used as a viable second-line complementary screening method.

## Data Availability

The data analyzed in this study is subject to the following licenses/restrictions: The data for this article are not publicly available because of privacy concerns. Requests to access these datasets should be directed to QZ, zhuqian_2009@163.com; HL, hongqian.liu@163.com.
